# Complementary and alternative therapies for precancerous lesions of gastric cancer: A protocol for a Bayesian network meta analysis

**DOI:** 10.1097/MD.0000000000024249

**Published:** 2021-01-15

**Authors:** Tianqi Zhang, Tiefeng Zhang, Chuancheng Li, Xixi Zhai, Qing Huo

**Affiliations:** a First College of Clinical Medicine, Shandong University of Traditional Chinese Medicine; b Second Affiliated Hospital of Shandong University of Traditional Chinese Medicine; c Affiliated Hospital of Shandong University of Traditional Chinese Medicine, Jinan, Shandong Province, China.

**Keywords:** Bayesian, complementary and alternative therapies, network meta-analysis, precancerous lesion of gastric cancer, protocol

## Abstract

**Background:**

Gastric cancer is one of the most common malignant tumors, which seriously affect peoples quality of life and threaten people's health. Precancerous lesions of gastric cancer (PLGC) are a critical stage in the occurrence and development of gastric cancer. Early effective intervention is an important means to prevent and control gastric cancer. In this study, we will evaluate the efficacy and safety of complementary and alternative therapies in the treatment of PLGC by Bayesian network meta-analysis (NMA).

**Methods:**

We will search PubMed, Cochrane Library, CNKI and other databases to gather randomized controlled trials (RCTs) on the treatment of PLGC with complementary and alternative therapies. Two reviewers will screen the literature and extract the data according to the inclusion and exclusion criteria, and then assess the quality and bias risk according to Cochrane's Risk of Bias Assessment Tool. Bayesian network meta-analysis will be conducted by Stata16.0 and WinBUGS1.4.3.

**Results:**

This study will compare and rank the efficacy and safety of different complementary and alternative therapies for PLGC.

**Conclusion:**

This study can provide reliable evidence for the efficacy and safety of complementary and alternative therapies in treatment of PLGC. We expect to provide scientific and rigorous evidence support for clinicians and patients, and then assist them to choose the optimum treatment.

**Protocol registration number:**

INPLASY2020120077.

## 1 Introduction

Gastric cancer is still a worldwide problem. In the latest global cancer statistics, the incidence of gastric cancer is the fifth in the world, and cancer-related mortality ranks third in the world.^[1,2]^ Although it is possible to identify and treat gastric cancer, most cases are diagnosed at an advanced stage. The cure rate of early gastric cancer is high, and the 5-year survival rate can reach over 90%.^[3]^ PLGC is mainly characterized by gastric mucosal dysplasia and intestinal metaplasia, which usually includes chronic atrophic gastritis, gastric polyps, gastric ulcer, residual gastritis and other diseases. Most potential patients do not attach much importance to understanding the prevention and treatment of PLGC. Timely intervention in PLGC stage is of great significance to delay the progress of the disease and even reversing the disease. Hence, early diagnosis and effective prevention and treatment of PLGC have proved to be an important topic for many medical workers.

Relevant study has confirmed that Helicobacter pylori infection is related to chronic gastritis, intestinal metaplasia and gastric cancer.^[4]^ Therefore, anti-Helicobacter pylori treatment should be carried out in time according to the doctors advice. Eradication of Helicobacter pylori infection can alleviate, control or delay gastric mucosal atrophy and intestinal metaplasia, and reverse gastric mucosal dysplasia. Endoscopic therapy is an effective means to reverse or even eradicate gastric mucosal dysplasia.^[5]^ At present, modern medicine still lacks drugs with clear curative effect and ideal treatment methods for PLGC, mainly including follow-up and monitoring, regular examination. Once severe dysplasia or even cancerous lesions are found, timely surgery or local gastric mucosal resection will be performed. In 2019, the latest European PLGC management consensus listed Helicobacter pylori eradication therapy as a treatment with high quality evidence.^[6,7]^ Other medical treatments include COX inhibitors, antioxidant, non-steroidal drugs, increasing mucosal nutrition and gastric mucosal protection, improving gastric motility, inhibiting bile reflux and other symptomatic treatments, and their efficacy remains to be discussed.^[7]^ But for chronic atrophic gastritis and precancerous lesions, there is no ideal intervention and treatment. Therefore, it is urgent to adopt various methods and standardized treatment to control its occurrence and development.

In recent years, complementary and alternative therapies have achieved prominent clinical effects in treating PLGC, which mainly include Chinese herbal medicine, acupuncture, massage, acupoint injection, psychotherapy, exercise therapy and so on. Modern pharmacological studies have shown that TCM has the functions of protecting gastric mucosa, resisting oxidation, regulating cell proliferation and regulating immunity.^[8,9]^ Besides, studies have shown that acupuncture and moxibustion has achieved satisfactory effects in improving symptoms by protecting gastric mucosa, regulating immunity and cell proliferation.^[10–12]^ What's more, acupoint application can make use of strengthening the gastric mucosal barrier, repairing gastric mucosal injury, regulating cell proliferation and apoptosis to prevent and treat atrophic gastritis.^[13]^ In addition, studies have manifested that drug therapy combined with appropriate psychotherapy can achieve pleasing effect in treating mood-related chronic gastric diseases.^[14]^ Through targeted psychological counseling, patients can have a correct understanding of the disease, eliminate their fear of cancer, build confidence in overcoming the disease, and better cooperate with doctors, so as to improve clinical curative effect and improve the quality of life of patients.^[15]^

In order to select the best treatment, this study will conduct NMA to compare these treatments. This study expands the total sample size by means of combining other relevant RCTs to minimize the bias. Significantly, Bayesian NMA is better than traditional analysis in comparing multiple treatments while the latter usually compares 2 interventions. Furthermore, NMA can combine direct and indirect comparisons, and therefore we can utilize it to select the optimal treatment for the patients with PLGC.

## 2 Methods

### 2.1 Study registration

This protocol has been registered on the international platform of registration system evaluation and meta-analysis agreement. (Registration number: INPLASY2020120077; URL= https://inplasy.com/inplasy-2020-12-0077/). We will conduct it strictly in accordance with the Preferred Reporting Items for Systematic Review and Meta-Analysis Protocols (PRISMA-P).^[16]^

### 2.2 Eligibility criteria

#### 2.2.1 Type of study

This study will contain all relevant peer-reviewed randomized controlled trials (RCTs). Review, animal study, case report, conference paper will not be included. The sample size of both treatment group and control group is at least 30. The language will be confined to Chinese or English.

#### 2.2.2 Participants

Patients who meet the diagnostic criteria of precancerous lesion of gastric cancer (PLGC); There are no restrictions in gender, age, race and nationality. Patients with other complicated and severe diseases will not be included.

#### 2.2.3 Interventions

PLGC patients in the treatment group are given complementary and alternative therapies, which include Chinese herbal medicine, acupuncture, massage, acupoint injection, psychotherapy, exercise therapy and so on. Different complementary and alternative therapies can be freely combined. In addition, they can be adopted based on routine western medicine or separately.

#### 2.2.4 Comparisons

The control group will adopt conventional western medicine, placebo, or other positive drugs. There is no restriction on the dosage form.

#### 2.2.5 Outcomes

1. The main outcomes: clinical efficacy rate, Helicobacter pylori infection clearance rate;
2. Secondary outcomes: the improvement rate of gastroscopy, the improvement rate of gastric mucosa pathology, Traditional Chinese Medicine Syndrome Score Scale (TCMSSS), the incidence of adverse events, economic costs.

### 2.3 Exclusion criteria

1. Repeated published literature;
2. Animal experiments, reviews, case reports, etc;
3. Combined with other complicated and severe diseases that have great interference in research;
4. Selected literature with different baseline data, incomplete data;
5. Literature with low quality.

### 2.4 Databases and search strategy

We will carefully discuss the details and precautions about literature retrieval, and then ascertain the search strategy after pre-retrieving literature. We will utilize a combination of MeSH and free-text terms. The retrieval time is from the establishment of the database to December 2020. All patients qualified in the trial are diagnosed as PLGC by clinical and endoscopic pathology. Search databases are as follows: PubMed, Cochrane Library, Cochrane Controlled Trial Center Registration, EMBASE, Web of Science, CNKI, Wanfang database, VIP database. In addition, we will proceed to follow up the literature in systematic review/meta-analysis. The search scheme of PubMed database is shown in Table 1.

**Table 1:** Search strategy for PubMed.

NO.	Search item
#1	gastric cancer [MeSH Terms] OR precancerous conditions [MeSH Terms]
#2	Stomach Neoplasms [Title/Abstract] OR precancerous lesion [Title/Abstract] OR atrophic gastritis [Title/Abstract] OR intestinal metaplasia [Title/Abstract] OR dysplasia [Title/Abstract] OR intraepithelial neoplasia [Title/Abstract]
#3	#1 OR #2
#4	Complementary Therapies [MeSH Terms]
#5	Complementary and alternative therapies [Title/Abstract] OR Alternative Medicine [Title/Abstract] OR Alternative Therapies [Title/Abstract] OR Complementary Medicine [Title/Abstract] OR Herbal therapy [Title/Abstract] OR Herb Therapy [Title/Abstract] OR Therapy, Alternative [Title/Abstract] OR Therapy, Complementary [Title/Abstract] OR Complementary Therapies [Title/Abstract] OR Therapies, Alternative [Title/ Abstract]
#6	#4 OR #5
#7	Chinese herbal medicines [Title/Abstract] OR Acupuncture [Title/Abstract] OR Moxibustion [Title/Abstract] OR Massage [Title/Abstract] OR Acupoint injection [Title/Abstract] OR psychotherapy [Title/Abstract] OR exercise [Title/Abstract]
#8	#6 OR #7
#9	Randomized Controlled Trial [Publication Type] OR Controlled Clinical Trial [Publication Type] OR Randomized [Title/Abstract] OR randomly [Title/Abstract] OR random allocation [Title/Abstract]
#10	#3 AND #8 AND #9

### 2.5 Data extraction

On the basis of the above strategy, we will retrieve all relevant literature from the database, and then import them into EndNote X9. Two researchers (Tianqi Zhang and Xixi Zhai) will browse the title and abstract separately, sort out the literature according to the inclusion and exclusion criteria, and then extract the data. Based on the previous step, qualified literature will be further searched. If the 2 researchers have different opinions, they will discuss and solve them or hand over to the third researcher (Chuancheng Li) for assistance. If the collected literature is incomplete, we will attempt to consult the corresponding author for help. The data of literature are extracted as follow:

1. Basic information: title, author, journal, year, trial registration number, country, etc.
2. Participants: gender, age, sample size, duration, etc.
3. Intervention: treatment method, frequency, dose, duration, etc.
4. Outcomes: The main outcomes include clinical efficacy rate, Helicobacter pylori infection clearance rate; Secondary outcomes include the improvement rate of gastroscopy, the improvement rate of gastric mucosa pathology, Traditional Chinese Medicine Syndrome Score Scale (TCMSSS), the incidence of adverse events, economic costs, etc.

### 2.6 Risk of bias analysis

The methodological quality will be evaluated by 2 researchers independently in the light of the Cochrane Collaboration's Risk of Bias Tool,^[17]^ which contains 7 items and every item is classified as “high” “unclear” and “low” levels.

### 2.7 Assessment of heterogeneity

We will utilize *I*^2^ statistics to assess the heterogeneity of each pairwise comparisons. When *I*^2^ < 50% and *P* > .10, we will adopt the fixed effect model since there is no significant heterogeneity. If not, we will perform a random effect model. Subgroup analysis and sensitivity analysis are needed for heterogeneity caused by factors such as trial design and treatment duration. In addition, supposing that the origin of heterogeneity is unaware, only descriptive analysis will be used.

#### 2.7.1 Subgroup analysis

Considering the heterogeneity, we will perform subgroup analysis in the light of the characteristics of research related to the source of heterogeneity. In addition, with regard to different design schemes, we will adopt subgroup analysis in accordance with age, gender, treatment type and course of disease.

#### 2.7.2 Sensitivity analysis

Sensitivity analysis will be assessed by excluding the literature one by one to ascertain whether the literature has an impact on heterogeneity. Once the heterogeneity of the study changes, this article may be the source of heterogeneity, and further analysis will be carried out, such as the difference in sample size and the reference standard of outcome indicators. If there is no obvious change before and after exclusion, it manifests that the results are stable and credible.

### 2.8 Statistical analysis

#### 2.8.1 Pairwise meta-analysis

We will execute STATA16.0 for pairwise meta-analysis. Continuous results will be summarized by SMD with 95% CI while dichotomous results will be analyzed by OR with 95% CI. *I*^2^ will be adopted to evaluate the heterogeneity of each pairwise comparisons.

#### 2.8.2 NMA

NMA will be implemented by STATA 16.0 and WinBUGS1.4.3 to synthesize direct and indirect evidence. The Bayesian NMA is mainly on the basis of the Markov-chain-Monte-Carlo (MCMC), so we will apply the MCMC in WinBUGS1.4.3 to execute Bayesian NMA of random effect model.^[18]^ After statistical analysis, the potential scale reduction factor (PSRF) is used to measure the convergence. When it is closer to 1, the convergence is much better, and the conclusion is more credible. Besides, we will adjust iteration times and annealing times based on the actual situation, and estimate the corresponding effective value of 95% CI. Moreover, this study will adopt surface under the cumulative ranking curve values to predict and rank the order of therapeutic effect. If the area under the curve is larger, the therapeutic effect is better. Furthermore, we will analyze the consistency of the main indicators. Supposing there is a closed loop, we will apply the node splitting method to estimate each loops inconsistency.

### 2.9 Publication bias

When talking about the potential publication bias, if each subgroup contains no less than 10 trials, this study will adopt funnel plot for analysis. The impact of publication bias can be evaluated by observing its symmetry. Assuming that the funnel chart is symmetrical, it demonstrates that there is no significant publication bias. Otherwise, it manifests that there may be publication bias, and then we will discuss the reasons.

### 2.10 Evaluation of evidence quality

In this study, GRADE method will be utilized to assess the quality of evidence and the strength of recommendations.^[19]^ The quality of the evidence means that people can decide the accuracy of the predicted value, which can be classified as high, medium, low and very low. The basic application principle of GRADE method in NMA mainly includes 5 aspects, namely, the research limitation, inconsistency, indirectness, publication bias and inaccuracy.

## 3 Discussion

PLGC is considered to be a crucial phase in the early diagnosis and prevention of intestinal gastric cancer.^[20]^ It takes a relatively long process for PLGC to develop into gastric cancer. Due to active follow-up monitoring and effective intervention, the development of gastric cancer is blocked, and the incidence and mortality of gastric cancer will be significantly reduced.^[21]^ Modern medical examination methods play an important role in the diagnosis of PLGC and early detection of lesions can be achieved.^[22,23]^ However, there is no ideal intervention and treatment measures for PLGC, and drug treatment is still controversial. Thus, complementary and alternative therapies are getting more and more attention.

Complementary and alternative therapies have achieved remarkable results in PLGC. As a comprehensive treatment method for PLGC, TCM is increasingly shown its advantages in treatment, reflecting the concept of “preventing disease”, and also the content of preventive medicine. With the guidance of TCM theory, TCM can intervene at multiple targets and levels to treat PLGC by protecting gastric mucosa, regulating immunity, affecting cell proliferation and differentiation, and anti-oxidation.^[24,25]^ Moreover, PLGC has gained more and more international recognition by treating it with TCM.^[26]^ Acupuncture and moxibustion have the characteristics of safety, simplicity, low cost and few side effects, and can significantly improve the clinical symptoms of patients.^[27]^ In addition, drug therapy combined with psychotherapy and exercise therapy can relieve patient psychological pressure, relieve their emotions, increase their confidence in overcoming the disease, and help improve the therapeutic effect of drugs, thus improving the quality of life of the patients.^[28]^

As we all know, traditional meta-analysis mainly focuses on the pairwise comparison of intervention measures, and it is impossible to conduct multiple comparative analysis of various intervention measures. NMA based on indirect comparative meta-analysis or multiple interventions has obvious advantages. Our study is the first network meta-analysis of complementary and alternative therapies interventions for PLGC. In this study, NMA was performed by comparing primary efficacy outcomes, secondary efficacy outcomes, safety indicators of all RCTs of complementary and alternative therapies in PLGC. Then the probability is further ranked based on these indicators. The goal of NMA is to select the best complementary and alternative therapies for PLGC.

Although NMA has many advantages, our study still has some limitations. First, although our research is based on literature rather than raw data, there may be false positives or false negatives. At the same time, there is a lack of strict random design for published complementary and alternative therapies for PLGC, and the quality of literature research is uneven, which also reduces the credibility of the research. Moreover, research literature is limited to Chinese and English, which also leads to some degree of deviation. We expect to include more rigorous, multi-center, large-sample, high-quality RCTs, so as to provide more convincing evidence, and then improve the level of evidence-based medicine. In any case, this study will provide reliable results for the best complementary and alternative therapies, and provide strong evidence for the significant advantages of these therapies in PLGC treatment.

## Author contributions

**Conceptualization:** Tianqi Zhang, Tiefeng Zhang, Qing Huo.

**Formal analysis:** Tianqi Zhang, Tiefeng Zhang.

**Methodology:** Chuancheng Li, Xixi Zhai.

**Project administration:** Tiefeng Zhang.

**Software:** Tianqi Zhang, Chuancheng Li.

**Writing – original draft:** Tianqi Zhang, Tiefeng Zhang.

**Writing – review & editing:** Tianqi Zhang, Tiefeng Zhang.

## Abbreviations

Abbreviations: CI = confidence interval, GRADE = grading of recommendations assessment development and evaluation, MeSH = medical subject headings, OR = odds ratio, PLGC = precancerous lesion of gastric cancer, RCT = randomized controlled trials, SMD = standardized mean difference.
